# The Sick, the Churches, and the Hospitals

**Published:** 1887-11-26

**Authors:** 


### THE SICK, THE CHURCHES, AND THE HOSPITALS.

A Conference in February.

Arrangements are in progress to hold a Conference of the clergy and ministers of all denominations (London and provincial) of distinguished laymen, the managers and officials of hospitals, medical practitioners, and others, early in February, 1888. Sir Andrew Clark, Bart., M.D., F.R.S., will explain his scheme to the Conference, and will be prepared to answer questions upon any points which may arise during the discussion. The Right Honourable the Earl of Northbrook, the Bishop of London, Lord Sandhurst, and many others have already arranged to be present, and names are coming in daily. A register has been opened at The Hospitals Association, Norfolk House, W.C., and anyone who desires to be present should forward name and address to the Secretary for registration. A first list of those who will attend will be published on the 10th December.

